# A Few Observations

**Published:** 1902-02

**Authors:** C. E. Boynton

**Affiliations:** Los Banos, Cal.


### CORRESPONDENCE.

### A FEW OBSERVATIONS.

Successful medical practice depends not altogether upon medical education, but somewhat upon dealing tactfully with human creatures. I will here set down a few observations along this line:

1. When consulting with a patient appear to have your mind upon him and his case only.

2. When asked how much is your bill for a call be ready to answer promptly $5.00, $10.00 and don't hesitate or stammer.

3. If your patient is new to you and the case is likely to require further attention, name your price and get your pay if future payment is likely to be an uncertainty.

4. If future payment is a certainty it is as well to book it, for then you may better hold your patient.

5. There are certain people that always get shocked at the size of their bills, yet it is dollars to you to hold their practice. Deal fairly with these people. If their bill is $10.00, ten dollars it remains if they are able to pay it.

6. If a man is both a kicker and slow to pay, give him to understand that the delicate nervous system of a physician is unhealthfully affected by financial parley, therefore, he being prone to this, you must require of him payment in advance.

7. Our worst customer is the man of lavish, unfulfilled promises. Don't let him "do you" but once.

8. When you have lost a patient by death and the party who is to pay you feels sore, present your bill in full and show your sympathy by a fair discount. But don't present a small bill in the first place.

9. A doctor is most respected when he is found with his books on attending his patients.

10. A curbstone doctor, a barroom doctor, a drug-store doctor, a farmer doctor, a hobby doctor and a social doctor—all of these are second-rate in the public eye as well as second-rate in fact.

C. E. Boynton, M.D.

Los Banos, Cal.

